# Glutathione alterations in depression: a meta-analysis and systematic review of proton magnetic resonance spectroscopy studies

**DOI:** 10.1007/s00213-024-06735-1

**Published:** 2024-12-21

**Authors:** Charles J. M. Bell, Mitul Mehta, Luwaiza Mirza, Allan H. Young, Katherine Beck

**Affiliations:** 1 Department of Forensic and Neurodevelopmental Science, Institute of Psychiatry, King’s College, Psychology & Neuroscience, London, UK; 2 South West London and St George’s NHS Foundation Trust, London, UK; 3 Girton College, Cambridge, UK; 4 Department of Neuroimaging, Institute of Psychiatry, King’s College, Psychology & Neuroscience, London, UK; 5 Department of Psychosis Studies, Institute of Psychiatry, King’s College, Psychology & Neuroscience, London, UK; 6 Department of Psychological Medicine, Institute of Psychiatry, King’s College, Psychology & Neuroscience, London, UK; 7 South London and Maudsley NHS Foundation Trust, London, UK

**Keywords:** GSH, Glutathione, Proton magnetic resonance spectroscopy, MRS, Major depressive disorder, MDD, Inflammation, Oxidative stress

## Abstract

**Background:**

Major depressive disorder (MDD) is a common and serious psychiatric disorder associated with significant morbidity. There is mounting evidence for the role of oxidative stress in the pathophysiology of depression.

**Objective:**

To investigate alterations in the brain antioxidant glutathione in depression by undertaking a meta-analysis of proton magnetic resonance spectroscopy (1H-MRS).

**Methods:**

MEDLINE, EMBASE and Psych Info databases were searched for case–control studies that reported brain glutathione levels in patients with depression and healthy controls. Means and variances (SDS) were extracted for each measure to calculate effect sizes. Hedges g was used to quantify mean differences. The Meta-analysis of Observational Studies in Epidemiology (MOOSE) and Preferred Reporting Items for Systematic Reviews and Meta-analyses (PRISMA) guidelines were followed.

**Results:**

8 studies that reported measurements for 230 patients with depression and 216 controls were included. Three studies included data for the occipital cortex and five studies for the medial frontal cortex. In the occipital cortex, GSH was lower in the patient group as compared to controls (g = -0.98, 95% [CI, -1.45—-0.50], P = < 0.001). In both the medial frontal cortex and in the combined all areas analysis there was no significant difference in GSH levels between cases and controls.

**Conclusions:**

This study found reduced levels of GSH specifically in the occipital region of patients with MDD. This provides some support for the role of oxidative stress in depression and suggests that targeting this system may provide future therapeutic opportunities. However, the meta-analysis was limited by the small number and quality of the included studies. More studies using high quality MRS methods in a variety of brain regions are needed in the future to test this putative hypothesis.

**Supplementary Information:**

The online version contains supplementary material available at 10.1007/s00213-024-06735-1.

## Introduction

Major depressive disorder (MDD) is a common and serious mood disorder that is thought to affect up to 3.8% of the population worldwide (around 280 million people) (Evaluation, 2021). MDD has a lifetime prevalence of 12% and is a leading cause of global disability (Filatova et al. 2021; Scott et al. 2022). It affects both a person’s mood and their ability to function and is associated with increased risk of suicide (Cavanagh et al. 2003).

The aetiology of MDD is complex, involving multiple risk factors including genetic and environmental factors (Wray et al. 2014). The neurochemistry of MDD is poorly understood with some studies suggesting a role for reduced serotonin function (Erritzoe et al. 2023) and proton magnetic resonance spectroscopy studies implicating glutamate abnormalities as well as increased levels of neuroinflammation in some patients (Filatova et al. 2021).

Effective and healthy functioning of brain tissue requires a balance between reactive oxygen species and antioxidants (Poljsak et al. 2013). Reactive oxygen species result in the formation of free radicals, highly reactive molecules which cause cellular and tissue damage in the form of oxidative stress. Antioxidants act by preventing this harmful effect, which may help to reduce neuroinflammation. Neuronal high oxygen use results in the brain being at increased risk from an imbalance in this system (Murray et al. 2024). In a number of studies of patients with MDD, evidence has been found of increased neuroinflammatory markers associated with lower levels of antioxidants (Morris et al. 2014; Osimo et al. 2020). Both reactive oxygen species and antioxidants are required for a healthy functioning system, and a tip in the balance towards increased oxidative stress has thus been linked to the development of depression (Correia et al. 2023; Bhatt et al. 2020).

Glutathione (GSH) is an important antioxidant found across species, including humans, and is the principal antioxidant in brain tissue (Dwivedi et al. 2020; Dringen 2000). GSH depletion has been associated with anxiety and stress-related pathologies (Zalachoras et al. 2020), and levels of GSH and associated enzymes are lower in the blood of patients with MDD (Maes et al. 2011; Stefanescu and Ciobica 2012; Kodydková et al. 2009; Rybka et al. 2013). A post-mortem study found lower levels of GSH in patients with MDD specifically in the prefrontal cortex (PFC) (Gawryluk et al. 2011), and furthermore anhedonia is negatively correlated with GSH levels in the occipital cortex in patients with MDD (Lapidus et al. 2014). These findings suggest that reduced levels of this antioxidant may be involved in the pathophysiology of major depressive disorder, and may prove a promising drug target (Maes et al. 2012).

Proton magnetic resonance spectroscopy (^1^H-MRS) has been used to determine the in vivo levels of GSH in different brain regions in patients with depression and controls. Given the ongoing interest in antioxidants and glutathione in particular as potential therapeutic targets in depression, we have undertaken a meta-analysis to summarise current work in this field and to highlight the contribution of glutathione to the antioxidant theory of depression.

Existing ^1^H-MRS studies are small and show some conflicting results, with different studies focusing on different regions of the brain. Given the prior evidence for lower GSH levels in patients with MDD, our hypothesis is that glutathione levels may be lower in patients compared to healthy controls and that these differences may be brain-region specific.

## Methods

### Selection procedures

A meta-analysis (PROSPERO ID: CRD42023376612) was performed according to the Meta-analysis of Observational Studies in Epidemiology (MOOSE) (Stroup et al. 2000) and Preferred Reporting Items for Systematic Reviews and Meta-analyses (PRISMA) (Liberati et al. 2009) frameworks. Three authors (K.B., C.B., and L.M.) independently searched MEDLINE, Embase, and PsychINFO from 1960 (or 1974 in the case of EMBASE) to March 30th 2023, identifying 159 papers in total (111 with duplicates removed). A further search was undertaken on 6th December 2023, which identified an additional 19 papers. The following keywords were used: ((MRS) or (magnetic resonance spectroscopy)) and ((depression) or (BPAD) or (bipolar affective disorder)) and ((GSH) or (glutathione)). Meta-analyses and systematic and narrative review articles were hand-searched for additional reports. Abstracts were screened, and the full texts of suitable studies were obtained. If full texts were not available, authors were contacted and full content was requested. Authors were also contacted when data was missing from the studies including GSH mean levels and standard deviation. Three authors (K.B., C.B., L.M.) selected the final studies included in the meta-analysis based on the following criteria. Data extraction was undertaken independently by two authors (C.B., L.M.).

### Selection criteria for the eta-analysis

Inclusion criteria were studies (1) including healthy participants, (2) utilising ^1^H-MRS to measure GSH levels in specified brain regions, (3) giving mean and SD for each group, (4) patients with depression only and not bipolar affective disorder in accordance with criteria specified in the Diagnostic and Statistical Manual for Mental Disorders (DSM) or the International Classification of Diseases (ICD-11), (5) information provided on sex of patients.

### Statistical analysis

All statistical analyses were carried out using the ‘metafor’ package (version 1.9–9) in the statistical programming language R (version 3.3.1). Mean and standard deviation values were extracted. A minimum of three studies was required for meta-analysis – the two brain regions identified with sufficient studies were the occipital cortex and the medial frontal cortex. This latter region included both the anterior cingulate cortex and the ventromedial prefrontal cortex as there is spatial overlap between these regions (Merritt et al. 2023). Standard effect sizes (Hedges’ g) for individual studies were estimated. The individual study effect sizes were then entered into a random effects meta-analytic model using restricted maximum likelihood estimation. I^2^ values were calculated to estimate between study heterogeneity. Funnel plots were visibly inspected for evidence of publication bias (Supplemental Figs. 1 and 2).

**Fig. 1:**
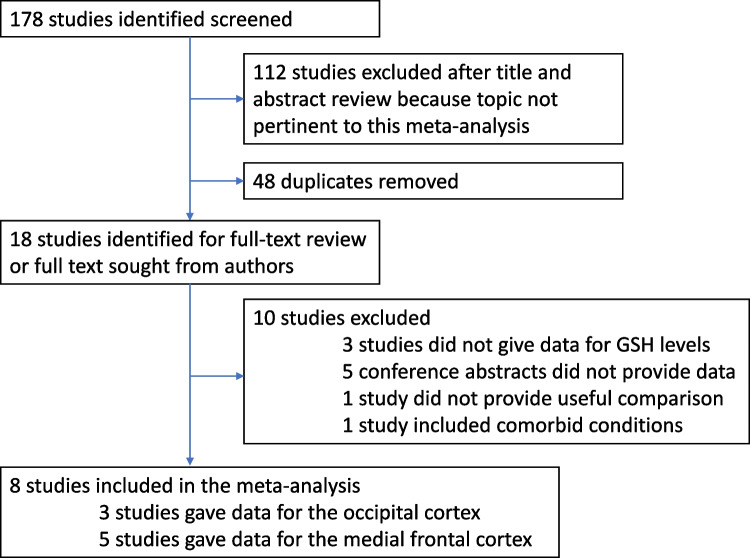
Search process summarising the review and exclusion of studies

**Fig. 2:**
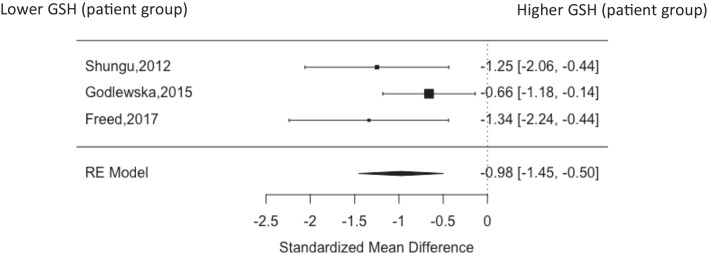
Forest plot of occipital cortex showing standardised mean difference between patients with MDD and healthy controls

### Risk of bias

Two researchers (L.M. and C.B.) assessed risk of bias in group matching based on variables using the Newcastle–Ottawa quality assessment for case–control studies (Stang 2010), and where there was a dispute a third senior supervising author checked the study information (Supplemental Table 1).

**Table 1:** Individual MRS studies with number of patients and demographics, MRS acquisition methodology and internal reference (PCr: phosophocreatine; Cr creatine)

Study	Brain region	Cases	HV	Cases mean age	Cases % male	HV mean age	HV % male	MRS acquisition methodology	MRS Internal reference
Shungu 2012	OCC	15	13	31.7 [9.6]	40	27.6 [7.4]	46.2	3 T EXCITE, J-edited spin echo difference	Water-scaled
Godlewska 2015	OCC	33	27	29.9 [10.6]	42.4	30.3 [10.6]	40.7	3 T SPECIAL	Cr and PCr
Taylor 2017	MFC and thalamus	17	18	22.5 [4.6]	35	23.9 [4.6]	61	7 T STEAM	Water-scaled
Freed 2017	OCC	19	8	15.6 [2.5]	57.8	16.1 [3.4]	37.5	3 T PRESS, J-edited spin echo difference, SPGR and FLAIR	Water-scaled
Hermens 2018	MFC and hipp	94	59	21.5 [2.8]	42.6	23.8 [2.9]	35.6	3 T PRESS	Not available
Draganov 2020	MFC	31	63	37.3 [10.8]	41.9	41.8 [10.1]	46.9	3 T PRESS	Water-scaled
Smith 2021	MFC and posterior cingulate	9	9	70 [7]	55.6	67 [7]	44.4	7 T STEAM	Cr and PCr
Tuura 2023	MFC	12	19	22.3 [2.8]	42%	27.5 [5.2]	37	3 T MEGAPRESS	Water-scaled

## Results

### Retrieved studies for the meta-analysis of GSH

The literature search identified a total of 8 studies involving 230 patients (mean age of 27, 43.5% male) and 216 healthy volunteers (mean age of 31.5, 42.8% male). Figure 1 shows the PRISMA flowchart. The seven included studies are summarized in Table 1. 17 studies were initially identified, but 10 studies were excluded due to lacking or unusable data. 3 studies referred to GSH but did not give data, 5 studies were conference abstracts – we were unable to gain access to this data, despite three attempts at email correspondence with the authors. One study split patients into suicide-attempters and non-attempters, and another reviewed depression traits in schizophrenia: both of these studies were excluded. 

Three studies contained data for more than one brain region: the results for these different brain regions were included in the final meta-analysis, giving a total of 11 studies in the final list of brain regions with data available. (Table 2) In total, there were three studies in the occipital region; five studies in the medial frontal cortex; and one each for the posterior cingulate cortex, hippocampus and thalamus. We therefore undertook separate meta-analysis for the occipital and medial frontal cortex.

**Table 2:** Combined and categorised MRS studies with number of patients and demographics

Brain region	Studies	Cases	HV	Effect size (95% CI)	Effect size p value	Heterogeneity I^2^%	Hetero P value	Cases average age	Cases % male	HV average age	HV % male
All	8	230	216	–	–	–	–	27.0	43.5	31.5	42.8
Combined	11*	350	302	−0.19 (−0.54 – 0.16)	0.285	75.3	0.0002	26.4	41.1	30.6	42.6
MFC	5	163	168	0.18 (−0.09 – 0.44)	0.1988	20.45	0.2302	27.3	42.4	32.7	43.2
OCC	3	67	48	−0.98 (−1.45—−0.50)	< 0.001	25.77	0.2996	26.3	46.2	27.2	41.7

^*^8 separate studies, but 3 with both MFC and OCC

## Occipital cortex

There were three studies using data from the occipital cortex including 67 patients (mean age of 26.3, 46.2% male) and 48 healthy volunteers (mean age of 27.2, 41.7% male) (Freed et al. 2017; Shungu et al. 2012; Godlewska et al. 2015). In this area GSH was decreased in the patient group (g = −0.98, 95% CI, −1.45—−0.50, P = < 0.001) (Fig. 2). Heterogeneity was 25.77% with no evidence of publication bias on visible inspection of the funnel plot (Supplemental Fig. 1).

## Medial frontal cortex

**Fig. 3:**
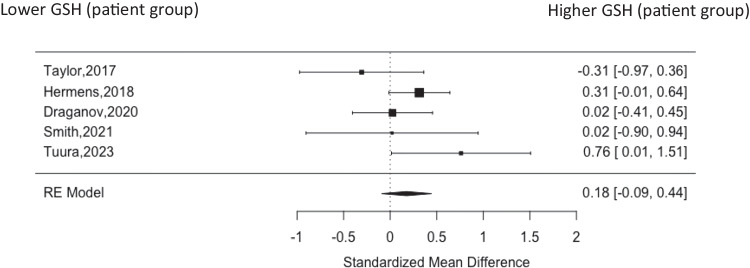
Forest plot of medial frontal cortex showing standardised mean difference between patients with MDD and healthy controls

Five studies included data for the medial frontal cortex (as defined in Methods) including 163 patients (mean age of 27.3, 42.4% male) and 168 healthy volunteers (mean age of 32.7, 43.2% male) (Taylor et al. 2017; Hermens et al. 2018; Draganov et al. 2020; Smith et al. 2021; Tuura et al. 2023). There was no significant finding for GSH levels in the medial frontal cortex (g = 0.18 (95% CI, −0.09–0.44)) (Fig. 3). Heterogeneity was 20.45% with no evidence of publication bias on visual inspection of funnel plot (Supplemental Fig. 2).

## Other brain areas

In addition to the areas highlighted above, there were also single studies available for the posterior cingulate cortex (Smith et al. 2021), hippocampus (Hermens et al. 2018), and thalamus (Taylor et al. 2017), all of which showed no difference in GSH levels between cases and controls. A forest plot showing all areas combined is available in Supplemental Fig. 3.

## Discussion

Our review found that people with MDD have lower GSH in the occipital cortex relative to healthy controls. This was not the case for the medial frontal cortex nor when an analysis was done including all areas. To our knowledge, this is the first meta-analysis to examine GSH in this patient group.

There is no post-mortem data available for the occipital cortex. However, a post-mortem study of the prefrontal cortex (Gawryluk et al. 2011) did show reduced GSH levels in patients with depression compared to controls. Whilst we did not find this replicated in our meta-analysis in this particular brain region (nor in any of the individual studies included), nonetheless we did find this difference in the occipital region.

In addition, in an in vivo study patients diagnosed with MDD, levels of anhedonia were associated with lower levels of GSH in the occipital cortex (Lapidus et al. 2014), suggesting that low GSH may be associated with particular symptoms of MDD. At present, the role of the occipital cortex in depression is not fully elucidated (Wu et al. 2023), although studies in other conditions suggest that it may play a role in oxidative stress (Zhang et al. 2021). GSH and similar antioxidant enzymes are lower in the blood of patients with MDD compared to controls, suggesting that there is an effect of GSH in the pathology of MDD (Maes et al. 2011; Stefanescu and Ciobica 2012; Kodydková et al. 2009; Rybka et al. 2013). It may therefore be that more ^1^H-MRS studies are required to more fully determine whether these post-mortem findings are replicated in vivo.

We found low levels of heterogeneity when analysis was done in individual brain regions, However when all areas were combined in the analysis the heterogeneity was much higher (I^2^ of 72.18). This may reflect differences across the studies including the duration of illness, age (in particular, the lower mean age of both healthy controls and MDD patients in the studies of occipital cortex), and medication use.

A meta-analysis of GSH has been done in patients with BPAD and found a significant increase in GSH in the anterior cingulate cortex in patients as compared with healthy controls (Das et al. 2019). In our study, we did not find a difference in the medial frontal cortex (which included the anterior cingulate cortex), and instead found the opposite effect in the occipital cortex. The authors of this meta-analysis in BPAD suggested that mood state may affect the cerebral GSH content, given similar effects seen with other antioxidant enzymes (Andreazza et al. 2007), and it may be that more granular studies are required that control for or investigate particular mood states and the impact on brain area specific GSH differences. This is particularly pertinent given the apparent increase in GSH in BPAD and reduction in unipolar depression, suggesting that the affective state may account for these differences. More studies are required to further investigate this hypothesis, and would offer the opportunity to measure GSH levels in different BPAD states such as psychosis.

Our results are consistent with the hypothesis that reduced GSH is associated with MDD, which supports the idea that oxidative stress may form part of the mechanism of the underlying MDD (Bhatt et al. 2020; Rodrigues et al. 2014). The loss of redox homeostatic balance in the brain in the presence of increased ROS or reduced GSH and similar antioxidant molecules may suggest a pro-inflammatory aetiology in MDD. GSH is thought to be the most important endogenous non-enzymatic antioxidant (Kurutas 2016) and hence may be a key target in future treatments for MDD. Given the implications of our study, further work in this area is likely to further illustrate the role of different parts of this system in maintaining a healthy balance between oxidative stress and antioxidants in neural tissue.

The main limitation of our meta-analysis is the small number of studies (together with small sample sizes) currently in the literature, with single studies only for the posterior cingulate cortex, the hippocampus, and the thalamus (none of which showed a difference in GSH levels between cases and controls). It is likely that further studies specifically in regions of the MFC and the OCC would be important in the future, particularly given the suggested importance of the former in post-mortem studies in depression. There is similarly in vivo evidence for the role of the occipital lobe, both structurally and functionally, in depression (Maller et al. 2014; Sanacora et al. 2002; Fernández et al. 2005; Guan et al. 2021). Further studies in this area will enable us to determine whether the current limited number of studies is confounding in any attempt to delineate location-specific differences in GSH.

Of note, all the studies undertaken in the OCC included in this meta-analysis used 3 T MRS strength, whereas there was variation in MRS strength across the other brain regions (7 T was used for two MFC studies, with 3 T used for three MFC studies). There was no consistent pattern of acquisition method used across studies. 7 T is likely to give a more precise reading for metabolites than 3 T generally in MRS studies (Pradhan et al. 2015; Ladd et al. 2018), although there is no clear guidance on this specifically for GSH measurement. There were two methods used for internal reference in the included studies, both phosphocreatine/creatine and water-scaling. There was no consistent pattern in the method used across the studies in the different brain regions. Studies which make use of phosphocreatinine/creatinine for this purpose may suffer from methodological error given the reported negative association between creatinine levels and depression scores (Faulkner et al. 2021). However, if such a negative association is indeed found, then such a technique is likely to give a higher ratio for GSH in MDD. Interestingly our results suggest, conversely, that GSH is lower in MDD.

Due to the low number of studies and the inconsistency in reporting variables across the studies, it was not possible to look at the association of GSH levels with sex or age, particular symptoms and medication use. Future studies will benefit from taking a more systematic approach in this area. Similarly, there was some lack of consistency in ^1^H-MRS approach and measurement, although this did not adversely affect our ability to compare across studies. Limited information was provided on symptom severity across studies. It is likely that controlling for these variables will offer a clearer picture of the trait vs state role of GSH, and in future pharmacological studies may additionally help in this regard.

## Conclusion

We found evidence for the role of lower GSH in patients with MDD in the occipital region of the brain, a difference which is not found in other brain regions or in combined brain region studies. However, the findings of this meta-analysis are limited by the small number of studies and MRS quality in some of the included studies. More studies are needed to explore this mechanism further, not least given the advent of medications targeting antioxidant systems which may be an important future therapeutic option in patients with MDD (Simon et al. 2023; Shuang et al. 2020).

## Supplementary Information

Below is the link to the electronic supplementary material.

Supplementary file1 (PPTX 289 KB)

## References

[CR1] Andreazza AC, Cassini C, Rosa AR et al (2007) Serum S100B and antioxidant enzymes in bipolar patients. J Psychiatr Res 41(6):523–529 16956621 10.1016/j.jpsychires.2006.07.013

[CR2] Bhatt S, Nagappa AN, Patil CR (2020) Role of oxidative stress in depression. Drug Discov Today 25(7):1270–1276 32404275 10.1016/j.drudis.2020.05.001

[CR3] Cavanagh JT, Carson AJ, Sharpe M et al (2003) Psychological autopsy studies of suicide: a systematic review. Psychol Med 33(3):395–405 12701661 10.1017/s0033291702006943

[CR4] Correia AS, Cardoso A, Vale N (2023) Oxidative stress in depression: the link with the stress response, neuroinflammation, serotonin, neurogenesis and synaptic plasticity. Antioxidants (Basel) 12(2):470. 10.3390/antiox12020470 36830028 10.3390/antiox12020470 PMC9951986

[CR5] Das TK, Javadzadeh A, Dey A et al (2019) Antioxidant defense in schizophrenia and bipolar disorder: A meta-analysis of MRS studies of anterior cingulate glutathione. Prog Neuropsychopharmacol Biol Psychiatry 91:94–102 30125624 10.1016/j.pnpbp.2018.08.006

[CR6] Draganov M, Vives-Gilabert Y, de Diego-Adeliño J et al (2020) Glutamatergic and GABA-ergic abnormalities in First-episode depression. A 1-year follow-up 1H-MR spectroscopic study. J Affect Disord 266:572–577 32056929 10.1016/j.jad.2020.01.138

[CR7] Dringen R (2000) Metabolism and functions of glutathione in brain. Prog Neurobiol 62(6):649–671 10880854 10.1016/s0301-0082(99)00060-x

[CR8] Dwivedi D, Megha K, Mishra R et al (2020) Glutathione in Brain: Overview of Its Conformations, Functions, Biochemical Characteristics, Quantitation and Potential Therapeutic Role in Brain Disorders. Neurochem Res 45(7):1461–1480 32297027 10.1007/s11064-020-03030-1

[CR9] Erritzoe D, Godlewska BR, Rizzo G et al (2023) Brain Serotonin Release Is Reduced in Patients With Depression: A [11C]Cimbi-36 Positron Emission Tomography Study With a d-Amphetamine Challenge. Biol Psychiat 93(12):1089–1098 36635177 10.1016/j.biopsych.2022.10.012

[CR10] Faulkner P, Paioni SL, Kozhuharova P et al (2021) Relationship between depression, prefrontal creatine and grey matter volume. J Psychopharmacol 35(12):1464–1472 34697970 10.1177/02698811211050550 PMC8652356

[CR11] Fernández A, Rodriguez-Palancas A, López-Ibor M et al (2005) Increased occipital delta dipole density in major depressive disorder determined by magnetoencephalography. J Psychiatry Neurosci 30(1):17–23 15644993 PMC543836

[CR12] Filatova EV, Shadrina MI, Slominsky PA (2021) Major depression: one brain, one disease, one set of intertwined processes. Cells 10(6):1283 34064233 10.3390/cells10061283 PMC8224372

[CR13] Freed RD, Hollenhorst CN, Weiduschat N et al (2017) A pilot study of cortical glutathione in youth with depression. Psychiatry Res Neuroimaging 270:54–60 29078101 10.1016/j.pscychresns.2017.10.001 PMC5673254

[CR14] Gawryluk JW, Wang JF, Andreazza AC et al (2011) Decreased levels of glutathione, the major brain antioxidant, in post-mortem prefrontal cortex from patients with psychiatric disorders. Int J Neuropsychopharmacol 14(1):123–130 20633320 10.1017/S1461145710000805

[CR15] Godlewska BR, Near J, Cowen PJ (2015) Neurochemistry of major depression: a study using magnetic resonance spectroscopy. Psychopharmacology 232(3):501–507 25074444 10.1007/s00213-014-3687-y PMC4302231

[CR16] Guan M, Liu X, Guo L et al (2021) Improved Pre-attentive Processing With Occipital rTMS Treatment in Major Depressive Disorder Patients Revealed by MMN. Front Hum Neurosci 15:648816 34234657 10.3389/fnhum.2021.648816 PMC8256852

[CR17] Hermens DF, Hatton SN, Lee RSC et al (2018) In vivo imaging of oxidative stress and fronto-limbic white matter integrity in young adults with mood disorders. Eur Arch Psychiatry Clin Neurosci 268(2):145–156 28357562 10.1007/s00406-017-0788-8

[CR18] Evaluation IoHMa (2021) Global Health Data Exchange (GHDx). Available at: http://ghdx.healthdata.org/gbd-results-tool?params=gbd-api-2019-permalink/d780dffbe8a381b25e1416884959e88b. Accessed 5 Nov 2024

[CR19] Kodydková J, Vávrová L, Zeman M et al (2009) Antioxidative enzymes and increased oxidative stress in depressive women. Clin Biochem 42(13–14):1368–1374 19527700 10.1016/j.clinbiochem.2009.06.006

[CR20] Kurutas EB (2016) The importance of antioxidants which play the role in cellular response against oxidative/nitrosative stress: current state. Nutr J 15(1):71 27456681 10.1186/s12937-016-0186-5 PMC4960740

[CR21] Ladd ME, Bachert P, Meyerspeer M et al (2018) Pros and cons of ultra-high-field MRI/MRS for human application. Prog Nucl Magn Reson Spectrosc 109:1–50 30527132 10.1016/j.pnmrs.2018.06.001

[CR22] Lapidus KA, Gabbay V, Mao X et al (2014) In vivo (1)H MRS study of potential associations between glutathione, oxidative stress and anhedonia in major depressive disorder. Neurosci Lett 569:74–79 24704328 10.1016/j.neulet.2014.03.056 PMC4108847

[CR23] Liberati A, Altman DG, Tetzlaff J et al (2009) The PRISMA statement for reporting systematic reviews and meta-analyses of studies that evaluate health care interventions: explanation and elaboration. J Clin Epidemiol 62(10):e1-34 19631507 10.1016/j.jclinepi.2009.06.006

[CR24] Maes M, Mihaylova I, Kubera M et al (2011) Lower whole blood glutathione peroxidase (GPX) activity in depression, but not in myalgic encephalomyelitis / chronic fatigue syndrome: another pathway that may be associated with coronary artery disease and neuroprogression in depression. Neuro Endocrinol Lett 32(2):133–140 21552194

[CR25] Maes M, Fišar Z, Medina M et al (2012) New drug targets in depression: inflammatory, cell-mediated immune, oxidative and nitrosative stress, mitochondrial, antioxidant, and neuroprogressive pathways. And new drug candidates—Nrf2 activators and GSK-3 inhibitors. Inflammopharmacology 20(3):127–150 22271002 10.1007/s10787-011-0111-7

[CR26] Maller JJ, Thomson RHS, Rosenfeld JV et al (2014) Occipital bending in depression. Brain 137(6):1830–1837 24740986 10.1093/brain/awu072

[CR27] Merritt K, McCutcheon RA, Aleman A et al (2023) Variability and magnitude of brain glutamate levels in schizophrenia: a meta and mega-analysis. Mol Psychiatry. 10.1038/s41380-023-01991-7 36806762 10.1038/s41380-023-01991-7 PMC10575771

[CR28] Morris G, Anderson G, Dean O et al (2014) The glutathione system: a new drug target in neuroimmune disorders. Mol Neurobiol 50(3):1059–1084 24752591 10.1007/s12035-014-8705-x

[CR29] Murray AJ, Humpston CS, Wilson M et al (2024) Measurement of brain glutathione with magnetic Resonance spectroscopy in Schizophrenia-Spectrum disorders — A systematic review and Meta-Analysis. Brain Behav Immun 115:3–12 37769980 10.1016/j.bbi.2023.09.017

[CR30] Osimo EF, Pillinger T, Rodriguez IM et al (2020) Inflammatory markers in depression: A meta-analysis of mean differences and variability in 5,166 patients and 5,083 controls. Brain Behav Immun 87:901–909 32113908 10.1016/j.bbi.2020.02.010 PMC7327519

[CR31] Poljsak B, Šuput D, Milisav I (2013) Achieving the balance between ROS and antioxidants: when to use the synthetic antioxidants. Oxid Med Cell Longev 2013:956792 23738047 10.1155/2013/956792 PMC3657405

[CR32] Pradhan S, Bonekamp S, Gillen JS et al (2015) Comparison of single voxel brain MRS AT 3T and 7T using 32-channel head coils. Magn Reson Imaging 33(8):1013–1018 26117693 10.1016/j.mri.2015.06.003 PMC4549223

[CR33] Rodrigues R, Petersen RB, Perry G (2014) Parallels between major depressive disorder and Alzheimer’s disease: role of oxidative stress and genetic vulnerability. Cell Mol Neurobiol 34(7):925–949 24927694 10.1007/s10571-014-0074-5 PMC4163504

[CR34] Rybka J, Kędziora-Kornatowska K, Banaś-Leżańska P et al (2013) Interplay between the pro-oxidant and antioxidant systems and proinflammatory cytokine levels, in relation to iron metabolism and the erythron in depression. Free Radic Biol Med 63:187–194 23707456 10.1016/j.freeradbiomed.2013.05.019

[CR35] Sanacora G, Mason GF, Rothman DL et al (2002) Increased occipital cortex GABA concentrations in depressed patients after therapy with selective serotonin reuptake inhibitors. Am J Psychiatry 159(4):663–665 11925309 10.1176/appi.ajp.159.4.663

[CR36] Scott F, Hampsey E, Gnanapragasam S et al (2022) Systematic review and meta-analysis of augmentation and combination treatments for early-stage treatment-resistant depression. J Psychopharmacol 37(3):268–278. 10.1177/02698811221104058 35861202 10.1177/02698811221104058 PMC10076341

[CR37] Shuang B, Wenliang G, Yangyang F et al (2020) Efficacy and safety of anti-inflammatory agents for the treatment of major depressive disorder: a systematic review and meta-analysis of randomised controlled trials. J Neurol Neurosurg Psychiatry 91(1):21 31658959 10.1136/jnnp-2019-320912

[CR38] Shungu DC, Weiduschat N, Murrough JW et al (2012) Increased ventricular lactate in chronic fatigue syndrome. III. Relationships to cortical glutathione and clinical symptoms implicate oxidative stress in disorder pathophysiology. NMR Biomed 25(9):1073–1087 22281935 10.1002/nbm.2772 PMC3896084

[CR39] Simon MS, Arteaga-Henríquez G, Fouad Algendy A et al (2023) Anti-Inflammatory Treatment Efficacy in Major Depressive Disorder: A Systematic Review of Meta-Analyses. Neuropsychiatr Dis Treat 19:1–25 36636142 10.2147/NDT.S385117 PMC9830720

[CR40] Smith GS, Oeltzschner G, Gould NF et al (2021) Neurotransmitters and Neurometabolites in Late-Life Depression: A Preliminary Magnetic Resonance Spectroscopy Study at 7T. J Affect Disord 279:417–425 33120242 10.1016/j.jad.2020.10.011 PMC8606178

[CR41] Stang A (2010) Critical evaluation of the Newcastle-Ottawa scale for the assessment of the quality of nonrandomized studies in meta-analyses. Eur J Epidemiol 25(9):603–605 20652370 10.1007/s10654-010-9491-z

[CR42] Stefanescu C, Ciobica A (2012) The relevance of oxidative stress status in first episode and recurrent depression. J Affect Disord 143(1–3):34–38 22840610 10.1016/j.jad.2012.05.022

[CR43] Stroup DF, Berlin JA, Morton SC et al (2000) Meta-analysis of Observational Studies in EpidemiologyA Proposal for Reporting. JAMA 283(15):2008–2012 10789670 10.1001/jama.283.15.2008

[CR44] Taylor R, Osuch EA, Schaefer B et al (2017) Neurometabolic abnormalities in schizophrenia and depression observed with magnetic resonance spectroscopy at 7 T. Bjpsych Open 3(1):6–11 28243459 10.1192/bjpo.bp.116.003756 PMC5288640

[CR45] Tuura RO, Buchmann A, Ritter C, Hase A, Haynes M, Noeske R, Hasler G (2023) Prefrontal glutathione levels in major depressive disorder are linked to a lack of positive affect. Brain Sci 13(10):1475 37891842 10.3390/brainsci13101475 PMC10605149

[CR46] Wray NR, Lee SH, Mehta D et al (2014) Research Review: Polygenic methods and their application to psychiatric traits. J Child Psychol Psychiatry 55(10):1068–1087 25132410 10.1111/jcpp.12295

[CR47] Wu F, Lu Q, Kong Y et al (2023) A Comprehensive Overview of the Role of Visual Cortex Malfunction in Depressive Disorders: Opportunities and Challenges. Neurosci Bull 39(9):1426–1438 36995569 10.1007/s12264-023-01052-7 PMC10062279

[CR48] Zalachoras I, Hollis F, Ramos-Fernández E et al (2020) Therapeutic potential of glutathione-enhancers in stress-related psychopathologies. Neurosci Biobehav Rev 114:134–155 32438253 10.1016/j.neubiorev.2020.03.015

[CR49] Zhang L, Huang J, Zhang Z, Cao Z (2021) Altered metabolites in the occipital lobe in migraine without aura during the attack and the interictal period. Front Neurol 12:656349 10.3389/fneur.2021.656349 PMC8172811 34093404

