# Supplementary material for: Glutathione alterations in depression: a meta-analysis and systematic review of proton magnetic resonance spectroscopy studies
Source: Psychopharmacology (Berl). 2024 Dec 21;242(4):717–24. doi: 10.1007/s00213-024-06735-1 (PMC11890406; doi:10.1007/s00213-024-06735-1)
Supplement: Supplementary file 1 — Supplementary file1 (PPTX 289 KB) [file 213_2024_6735_MOESM1_ESM.pptx]

## Slide 1
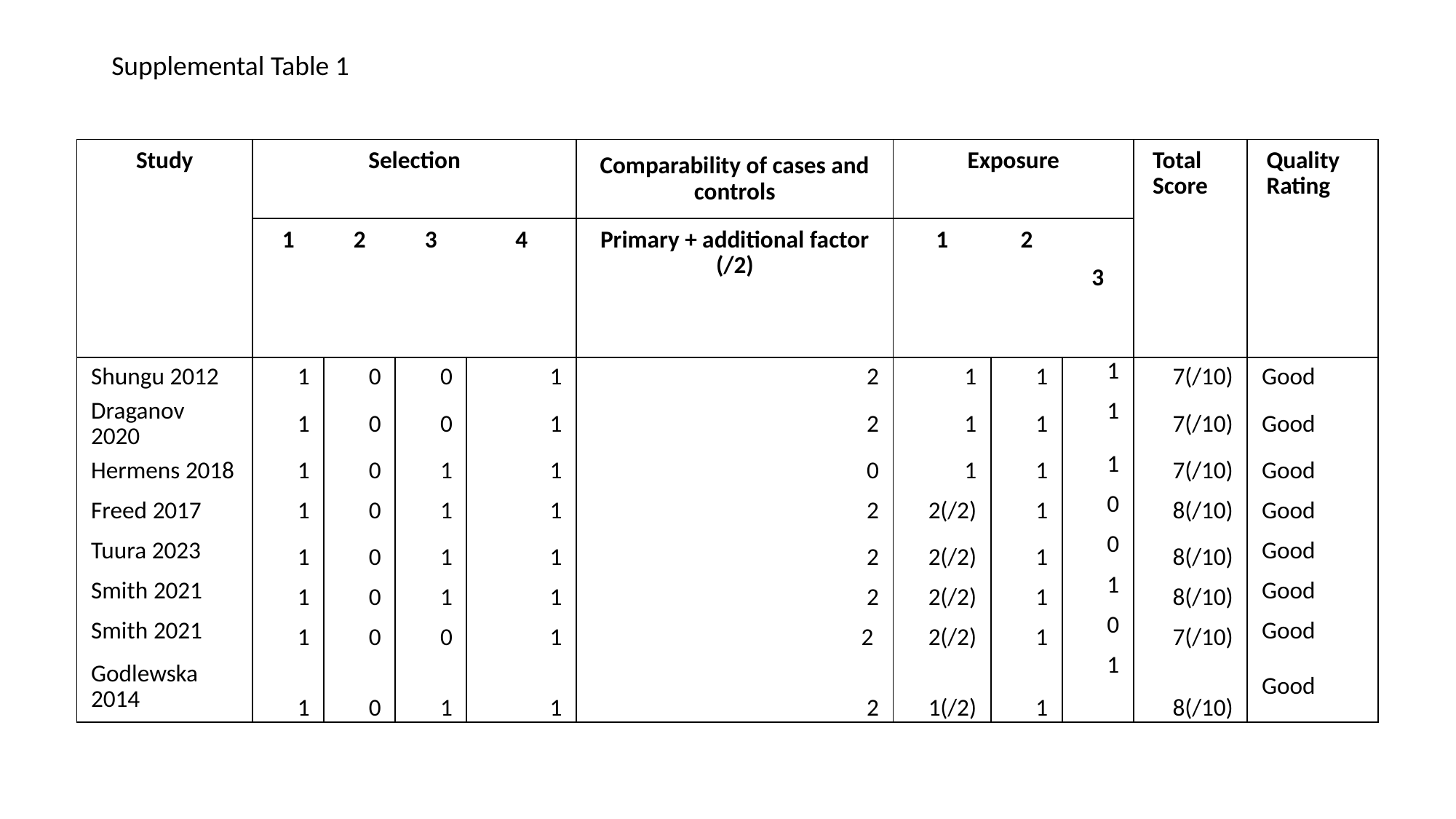

Supplemental Table 1
| Study | Selection | | | | Comparability of cases and controls | Exposure | | | Total Score | Quality Rating |
| --- | --- | --- | --- | --- | --- | --- | --- | --- | --- | --- |
| | 1 | 2 | 3 | 4 | Primary + additional factor (/2) | 1 | 2 | 3 | | |
| | | | | | | | | | | |
| Shungu 2012 | 1 | 0 | 0 | 1 | 2 | 1 | 1 | 1 | 7(/10) | Good |
| Draganov 2020 | 1 | 0 | 0 | 1 | 2 | 1 | 1 | 1 | 7(/10) | Good |
| Hermens 2018 | 1 | 0 | 1 | 1 | 0 | 1 | 1 | 1 | 7(/10) | Good |
| Freed 2017 | 1 | 0 | 1 | 1 | 2 | 2(/2) | 1 | 0 | 8(/10) | Good |
| Tuura 2023 | 1 | 0 | 1 | 1 | 2 | 2(/2) | 1 | 0 | 8(/10) | Good |
| Smith 2021 | 1 | 0 | 1 | 1 | 2 | 2(/2) | 1 | 1 | 8(/10) | Good |
| Smith 2021 | 1 | 0 | 0 | 1 | 2 | 2(/2) | 1 | 0 | 7(/10) | Good |
| Godlewska 2014 | 1 | 0 | 1 | 1 | 2 | 1(/2) | 1 | 1 | 8(/10) | Good |

## Slide 2
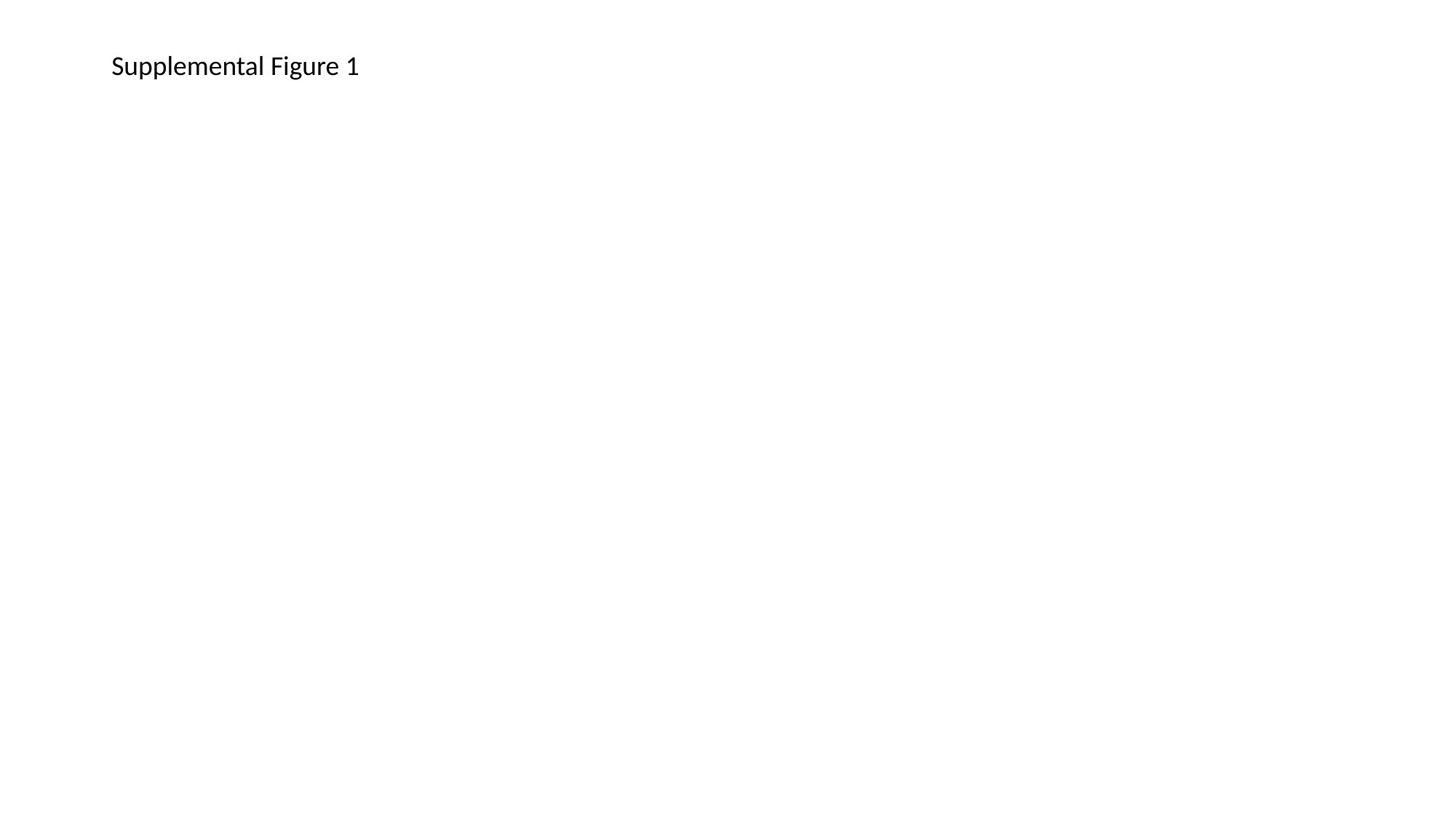

Supplemental Figure 1

## Slide 3
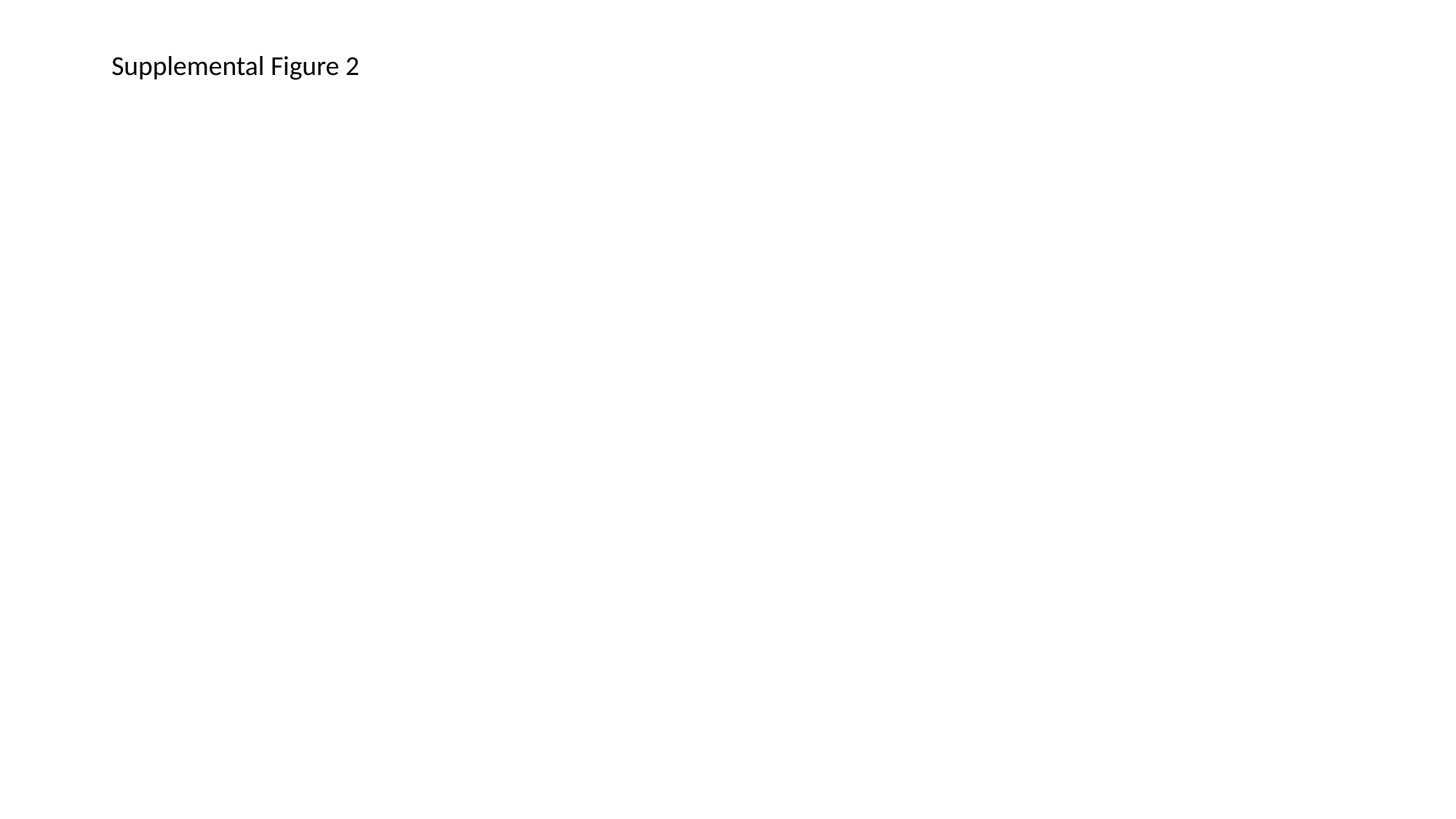

Supplemental Figure 2

## Slide 4
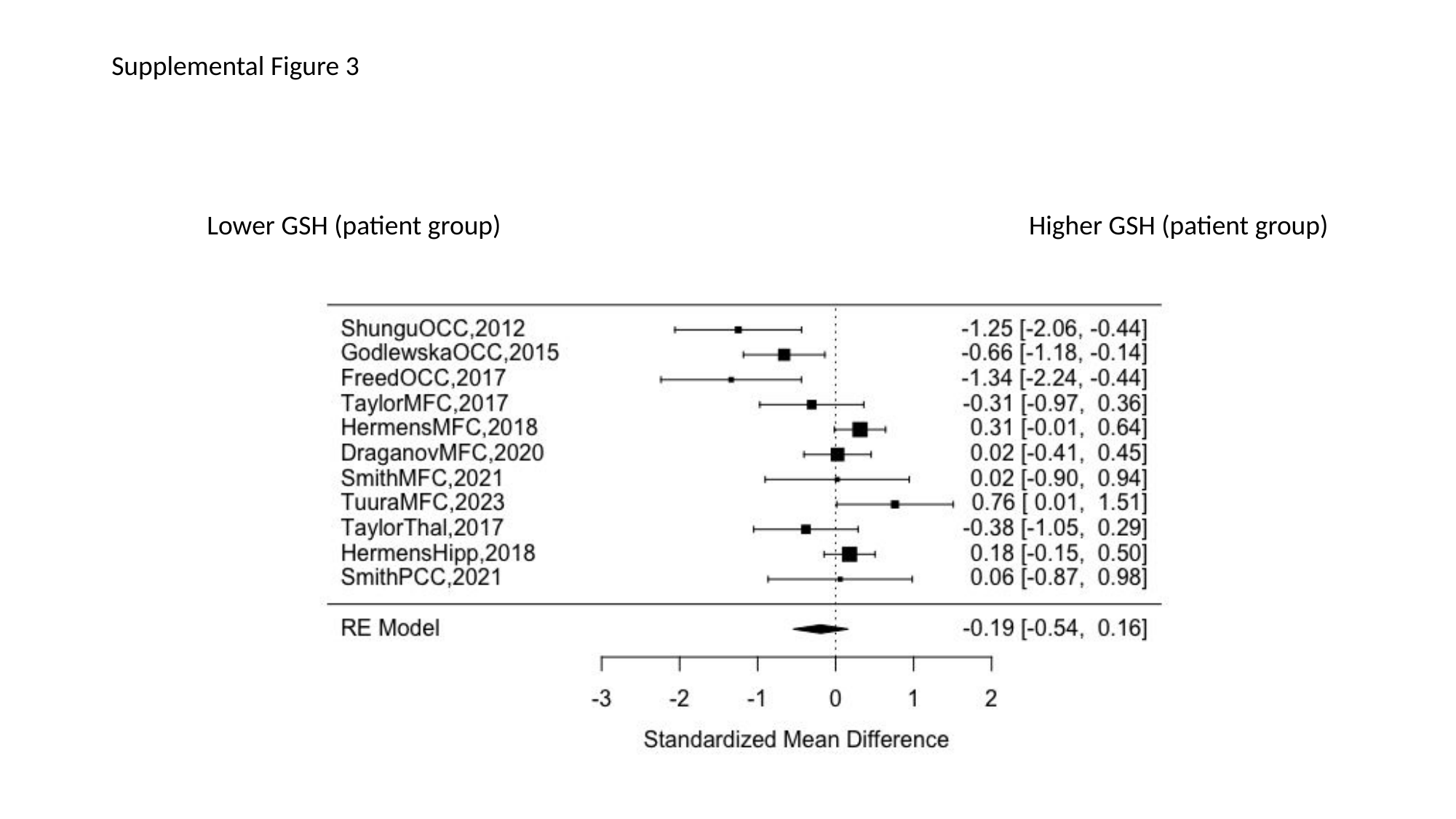

Supplemental Figure 3
Lower GSH (patient group)
Higher GSH (patient group)
